# Biological role and clinical value of miR‐99a‐5p in head and neck squamous cell carcinoma (HNSCC): A bioinformatics‐based study

**DOI:** 10.1002/2211-5463.12478

**Published:** 2018-06-26

**Authors:** Yu‐ting Chen, Jian‐ni Yao, Yu‐tao Qin, Kai Hu, Fang Wu, Ye‐ying Fang

**Affiliations:** ^1^ Department of Pathology First Affiliated Hospital of Guangxi Medical University Nanning Guangxi China; ^2^ Department of Radiation Oncology Radiation Oncology Clinical Medical Research Center of Guangxi First Affiliated Hospital of Guangxi Medical University Nanning Guangxi China

**Keywords:** expression, head and neck squamous cell carcinoma, miR‐99a‐5p, target genes

## Abstract

MicroRNAs (miRNAs) are confirmed to be tumor promoters or suppressors in multiple squamous cell carcinomas (SCCs). miR‐99a‐5p has been demonstrated to be downregulated in cancerous tissues, but its functional role in head and neck SCC (HNSCC) and its mechanism of action have not been fully elucidated. Here, we studied the expression of miR‐99a‐5p in HNSCC and performed a clinical value assessment and then extracted mature expression data from The Cancer Genome Atlas (TCGA) and microarrays from Gene Expression Omnibus (GEO). Furthermore, biological analysis was constructed via online prediction tools. The results revealed that miR‐99a‐5p expression was markedly lower in HNSCC tissues than in normal tissues, which also showed significance in the prognosis of HNSCC. However, its diagnostic value could not be verified due to the lack of body fluid samples. Additionally, miR‐99a‐5p was expressed at higher levels in patients with low histological grade neoplasms than those with high histological grade neoplasms. The age of the patient might also be a possible clinical parameter affecting miR‐99a‐5p expression. Furthermore, miR‐99a‐5p significantly influenced HNSCC progression by regulating the PI3K‐Akt signaling pathway, in which the key target genes were upregulated in 519 HNSCC tissues compared to 44 normal tissues, as determined by the Gene Expression Profiling Interactive Analysis (GEPIA). In conclusion, our study may provide insights into the expression and mechanism of miR‐99a‐5p in HNSCC. Further studies are required to elucidate the role of miR‐99a‐5p and its potential clinical applications for HNSCC.

**Abbreviations**
BP: biological process
CC: cellular component
DAVID: Database for Annotation, Visualization, and Integrated Discovery
FN: false negative
FP: false positive
GEO: Gene Expression Omnibus
GEPIA: Gene Expression Profiling Interactive Analysis
GO: gene ontology
HNSCC: head and neck squamous cell carcinoma
IGF1R: insulin‐like growth factor 1 receptor
KEGG: Kyoto Encyclopedia of Genes and Genomes
LR: likelihood ratio
MF: molecular function
MTOR: mechanistic target of rapamycin
PDGFRB: platelet‐derived growth factor receptor, beta polypeptide
PIK3CD: phosphatidylinositol‐4,5‐bisphosphate 3‐kinase catalytic subunit data
PPI: protein–protein interaction
ROC: receiver operating characteristic
SCC: squamous cell carcinoma
TCGA: The Cancer Genome Atlas
TN: true negative
TNM: tumor, node, and metastasis
TP: true positive

Squamous cell carcinomas (SCCs), also known as epidermoid carcinomas, are cancers that derived from squamous epithelial cells, which occur in the head and neck, thyroid, esophagus, lung, penis, prostate, bladder, vagina, and cervix 1, 2, 3, 4, 5, 6, 7, 8, 9. Of these, head and neck SCC (HNSCC) has attracted the attention of researchers due to its significant etiology including tobacco 10, alcohol 11, and human papilloma virus infection 12 associated with people's lifestyles. HNSCCs, the most frequent head and neck neoplasms, are originating from squamous cells in the nasal and oral cavity, paranasal sinuses, pharynx, larynx, and salivary glands. Men are at a higher risk of HNSCC than women. In particular, cancers of the oral cavity and pharynx were reported to cause 49 670 new cases and 9700 deaths worldwide in 2017 and were the ninth highest cause of new cancer cases in men.

A deeper understanding of HNSCC is accompanied with some remarkable explorations for diagnosis, prognosis, and potential pathogenesis 1, 13, 14, 15, 16, 17. Current treatment trends include targeted therapy combined with essential chemotherapy, radiotherapy, or immunotherapy 18, 19, 20, 21, 22, 23. Despite this progress, the increasing morbidity, mortality, and complex pathological changes of HNSCC urgently necessitate more effective means for its diagnosis and treatment, especially targeted treatments based on the further exploration of novel biomarkers.

MicroRNAs (miRNAs) are small noncoding RNAs with 21–25 nucleotides, which have been confirmed to be involved in the initiation and development of multiple SCCs 24, 25, 26, 27, 28, 29. Studies have shown significantly aberrant expression of several miRNAs in HNSCCs 27, 30, 31, 32, 33, indicating that miRNA expression levels may be valuable for the clinical diagnosis and prognosis of HNSCC. There is a strong need to characterize the clinical application of miRNAs for HNSCC. Among validated miRNAs, miR‐99a‐5p, the major member of miR‐99a family, has been demonstrated to be associated with carcinogenesis and deterioration in several cancers such as breast cancer, endometrial carcinoma, osteosarcoma, bladder cancer, lung adenocarcinoma, and hepatocellular carcinoma 34, 35, 36, 37, 38, 39. Several genes have been found to be regulated by miR‐99a‐5p, which is also enriched in relevant biological pathways 35, 40, 41, 42, 43, 44, 45.

In HNSCCs, miR‐99a‐5p has been reported to be downregulated in cancerous tissues 46, 47. Nevertheless, its functional role and relevant mechanism remain to be fully elucidated. In this study, based on the data acquired from Gene Expression Omnibus (GEO), The Cancer Genome Atlas (TCGA), and relevant literature, and using prediction tools (Fig. 1), we calculated the expression level and clinical value of miR‐99a‐5p, and performed biological analysis. This study might provide a comprehensive explanation of the clinical value and underlying mechanism of miR‐99a‐5p in HNSCC, to identify abnormally expressed miRNAs involved in HNSCC.

**Figure 1:**
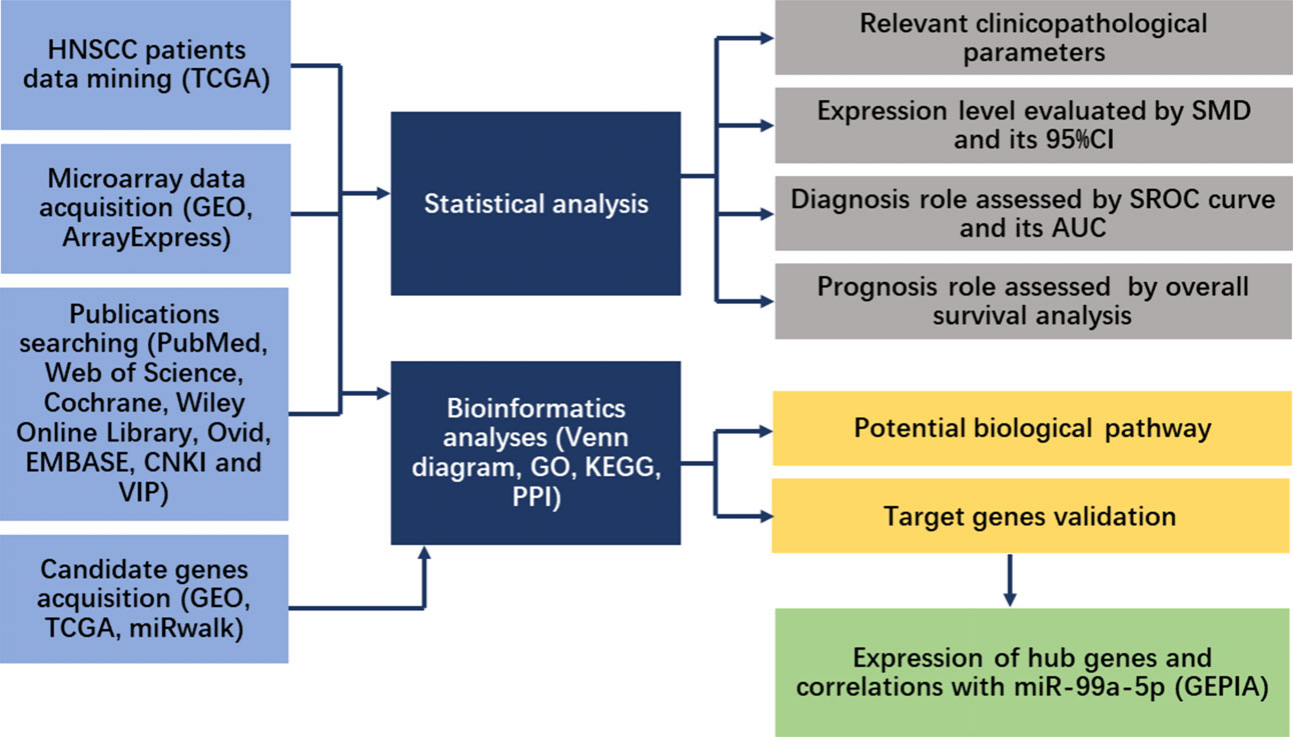
Flowchart of the study design.

## Materials and methods

### TCGA data in HNSCC patients

Mature expression data of miR‐99a‐5p in HNSCC and clinical information were obtained from TCGA datasets via UCSC (http://xena.ucsc.edu/; Accession Number: MIMAT0000097). The IIIuminaHiseq platform included 483 HNSCC patients and 44 adjacent noncancerous head and neck tissues, while the IIIuminaGA platform included 36 patients with HNSCC. No further transformation was performed for the expression data. We explored the possible association between miR‐99a‐5p expression and clinical parameters for HNSCC patients using the two platforms together or the IIIuminaHiseq platform alone, for further comparison of these two approaches. Based on TCGA data, the diagnostic and prognostic significance of miR‐99a‐5p was evaluated using the receiver operating characteristic (ROC) curve and the Kaplan–Meier curve, respectively.

### Microarray data acquisition and extraction

We obtained available miRNA expression profiling of HNSCC from the GEO database (http://www.ncbi.nlm.nih.gov/geo/) and ArrayExpress (https://www.ebi.ac.uk/arrayexpress/). The search terms were as follows: (‘head AND neck’ OR ‘laryngeal’ OR ‘salivary gland’ OR ‘lip’ OR ‘mouth’ OR ‘tongue’ OR ‘nasopharyngeal’ OR ‘pharyngeal’ OR ‘OSCC’ OR ‘oral squamous cell’ OR ‘laryngeal’ OR ‘HNSCC’) AND (‘carcinoma’ OR ‘tumor’ OR ‘cancer’ OR ‘neoplas*’ OR ‘malignan*’). Microarray datasets were eligible with the entry criteria listed below: (1) Patients in each dataset were diagnosed with HNSCC; (2) both cancerous and noncancerous specimens were included in each dataset with a sample size of no less than three per group; and (3) miR‐99a‐5p expression data should be provided. Several relevant elements were extracted from the microarray datasets: author, publication year, country, platform, sample size, and miR‐99a‐5p expression level. Two authors (Yu‐ting Chen and Jianni Yao) independently extracted essential information from all selected chips. Conflicting opinions were solved by a discussion.

In addition, we searched the PubMed, Web of Science, Cochrane, Wiley Online Library, Ovid, EMBASE, CNKI, and VIP databases for relevant articles. The following strategy was constructed for searching: (microRNA‐99 OR hsa‐mir‐99 OR miR‐99 OR MIRN99a microRNA OR microRNA‐99a OR miR‐99a OR hsa‐mir‐99a OR MIRN99A OR mir‐99a) AND (‘head AND neck’ OR ‘laryngeal’ OR ‘salivary gland’ OR ‘lip’ OR ‘mouth’ OR ‘tongue’ OR ‘nasopharyngeal’ OR ‘pharyngeal’ OR ‘OSCC’ OR ‘oral squamous cell’ OR ‘laryngeal’ OR ‘HNSCC’) AND (‘carcinoma’ OR ‘tumor’ OR ‘cancer’ OR ‘neoplas*’ OR ‘malignan*’). Studies that provided case numbers, mean, and standard deviation (SD) were included.

### Statistical analysis

Statistical analyses were performed using SPSS 23.0 (IBM, NY, USA) and Stata version 12.0. Scatter diagrams were plotted for each study using GraphPad Prism 7.0. We also used SPSS 23.0 to calculate the mean ± SD for all the studies based on the expression value of miR‐99a‐5p. Stata version 12.0 was used to perform continuous variable meta‐analysis by evaluating the overall SMD and 95% CI. Both fixed‐effect and random‐effect model were employed, while the heterogeneity was analyzed by chi‐square and *I* ^*2*^ tests. Sensitivity analysis was added to explain the heterogeneity. Results were considered statistically significant if the observed SMD with 95%CI did not cross 0. Additionally, we constructed Begg's funnel and Egger's plot to detect publication bias.

For diagnostic tests, we used SPSS 23.0 to plot the ROC curve and to calculate the true positive (TP), false positive (FP), false negative (FN), and true negative (TN) for each included study. Then, diagnosis meta‐analysis was performed via MetaDisc 1.4. Sensitivity, specificity, positive likelihood ratio (+LR), negative likelihood ratio (−LR), and diagnostic odds ratio (OR), as well as the summarized ROC curve (SROC), were chosen to describe the possible diagnostic value of miR‐99a‐5p for HNSCC. For practical application, we made a conclusion via the overall consideration of our diagnosis test results and the provided body fluid samples.

### Bioinformatics analyses

To predict the putative target genes of miR‐99a‐5p, we acquired candidate genes from http://www.ncbi.nlm.nih.gov/geo/query/acc.cgi?acc=GSE85614 (log2FC < 0), TCGA database (log2FC > 1 and *P* < 0.05). The miRwalk 2.0, which included miRWalk, Targetscan, miRanda, miRDB, miRNAMap, miRBridge, RNA22, miRMap, PITA, RNAhybrid, PicTar, and Microt4, was also applied to selected genes with a computer algorithm. Genes overlapping at least two prediction platforms were selected. Based on the above source, prospective genes were screened through intersection by online tools (http://bioinformatics.psb.ugent.be/webtools/Venn/). Meanwhile, validated genes from publications were also added.

Based on the predicted target genes, we conducted Gene ontology (GO) and Kyoto Encyclopedia of Genes and Genomes (KEGG) pathway analysis using online tools (https://david.ncifcrf.gov/) to determine the underlying mechanism of miR‐99a‐5p in HNSCC. The STRING database (https://string-db.org/) was also utilized to construct a PPI network for further characterizing the interactions among promising target genes of miR‐99a‐5p. Furthermore, hub genes with over five degrees were selected. In addition, we acquired differentially expressed genes of HNSCC from the Gene Expression Profiling Interactive Analysis (GEPIA) (|log2FC| > 1.5, *P* < 0.05) and conducted another KEGG pathway analysis to detect the potential pathways for the progression of HNSCC.

### Expression of hub genes and their correlations with miR‐99a‐5p

Based on GEPIA 48, we detected the expression of hub genes in HNSCC and normal tissues to further identify the target genes of miR‐99a‐5p. We also performed Spearman's correlation analysis to explain the correlation between hub genes and miR‐99a‐5p. Besides, the protein level of those hub genes was acquired from The Human Protein Atlas.

## Results

### Relationships between miR‐99a‐5p expression and clinicopathological parameters in HNSCC

**Figure 2:**
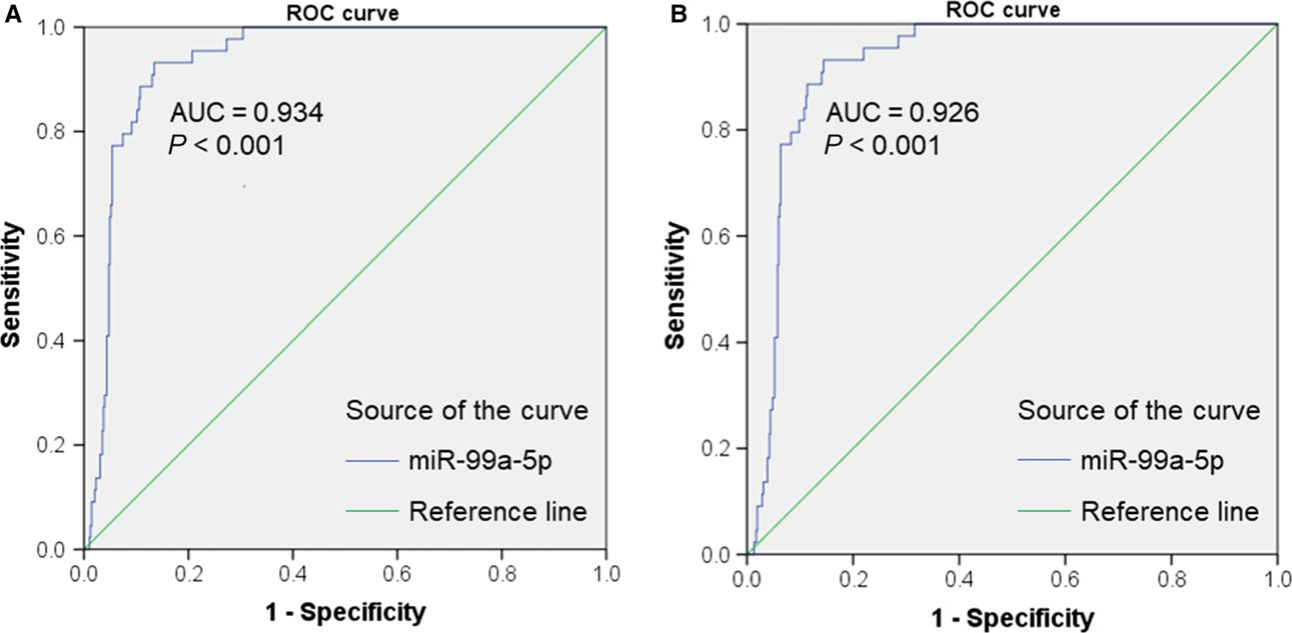
Receiver operating characteristic (ROC) curves of miR‐99a‐5p in HNSCC based on TCGA data. (A) Diagnostic value of miR‐99a‐5p for HNSCC based on the IIIuminaHiseq platform (AUC = 0.934, *P* < 0.001). (B) Diagnostic value of miR‐99a‐5p for HNSCC based on the IIIuminaHiseq and IIIuminaGA platforms (AUC = 0.926, *P* < 0.001).

**Figure 3:**
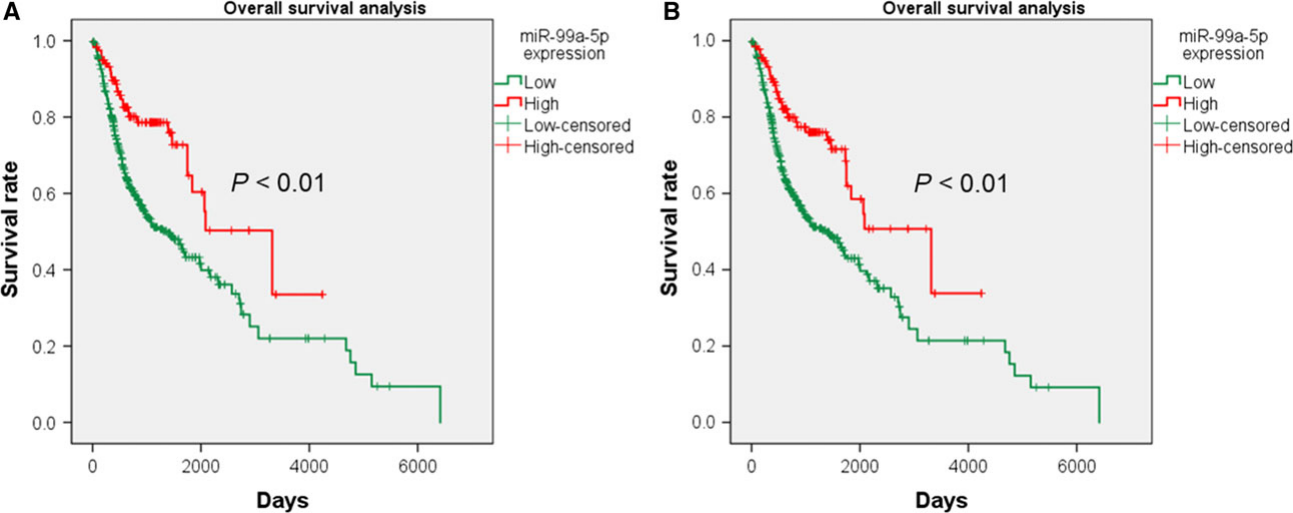
Kaplan–Meier curves of different miR‐99a‐5p expression levels based on TCGA data. (A) The overall survival of HNSCC patients varies with different miR‐99a‐5p expression levels based on the IIIuminaHiseq platform (*P* < 0.01). (B) The overall survival of HNSCC patients varies with different miR‐99a‐5p expression levels based on the IIIuminaHiseq and IIIuminaGA platforms (*P* < 0.01).

Statistical analysis based on the IIIuminaHiseq platform (Table 1) revealed that miR‐99a‐5p was expressed at a lower level in HNSCC tissues than in normal tissues (7.987 ± 1.467 vs 10.348 ± 0.625, respectively; *P* < 0.001). In addition, miR‐99a‐5p was expressed at higher levels in G1–G2 than in G3–G4 neoplasms (8.140 ± 1.239 vs 7.968 ± 1.525, respectively, *P* = 0.001). When statistical analysis was carried out using a combination of the IIIuminaHiseq and IIIuminaGA platforms (Table 2), the results revealed that miR‐99a‐5p was expressed at lower levels in HNSCC tissues than in adjacent normal tissues (8.028 ± 1.498 vs 10.348 ± 0.625, respectively, *P* < 0.001). Significant differences were also observed among neoplasms of different histological grades (7.841 ± 1.410 vs 8.413 ± 1.622, respectively, *P* < 0.001). In addition, miR‐99a‐5p expression was higher in patients over 50 years than in those less than 50 years (8.090 ± 1.453 vs 7.691 ± 1.695, respectively, *P* = 0.027). As for the diagnostic test based on TCGA, miR‐99a‐5p might show significant diagnostic value for HNSCC (AUC = 0.934, *P* < 0.001; AUC = 0.926, *P* < 0.001; Fig. 2). However, the tissue types of patients were unknown. Additionally, survival analysis indicated a probable prognostic value for HNSCC patients (*P* < 0.01; Fig. 3). The added IIIuminaGA platform did not significantly affect our research; nevertheless, it reminds us of the need for more samples for further exploration of the relationships between miR‐99a‐5p expression and clinicopathological parameters of HNSCC patients.

**Table 1:** Relationships between the expression value of miR‐99a‐5p and clinicopathological parameters in HNSCC patients based on the IIIuminaHiseq platform

Clinicopathological features		*n*	miR‐99a‐5p expression level	*P* value
Tissue	Noncancerous	44	10.348 ± 0.625	< 0.001
	Cancerous	483	7.987 ± 1.467	
Gender	Male	351	8.046 ± 1.515	0.152
	Female	132	7.831 ± 1.323	
Age	≥ 50	405	8.040 ± 1.424	0.061
	< 50	77	7.698 ± 1.660	
T	T1–T2	172	8.092 ± 1.477	0.05
	T3–T4	251	7.817 ± 1.371	
N	N0	163	8.005 ± 1.374	0.308
	N1–N3	227	7.854 ± 1.482	
M	M0	174	8.008 ± 1.482	0.139
	M1	1		
Stage	I–II	109	8.140 ± 1.239	0.282
	III–IV	361	7.968 ± 1.525	
Histologic grade	G1–G2	341	7.816 ± 1.393	0.001
	G3–G4	122	8.332 ± 1.582	
Lymphovascular invasion	Yes	113	7.968 ± 1.458	0.180
	No	211	7.747 ± 1.388	
Alcohol	Yes	319	8.046 ± 1.445	0.194
	No	156	7.859 ± 1.521	

**Table 2:** Relationships between the expression value of miR‐99a‐5p and clinicopathological parameters in HNSCC patients based on the IIIuminaHiseq and IIIuminaGA platforms

Clinicopathological features		*n*	miR‐99a‐5p expression level	*P* value
Tissue	Noncancerous	44	10.348 ± 0.625	0.001
	Cancerous	519	8.028 ± 1.498	
Gender	Male	379	8.079 ± 1.558	0.198
	Female	140	7.889 ± 1.317	
Age	≥ 50	436	8.090 ± 1.453	0.027
	< 50	82	7.691 ± 1.695	
T	T1 ~ T2	184	8.097 ± 1.475	0.117
	T3 ~ T4	272	7.882 ± 1.402	
N	N0	175	8.034 ± 1.386	0.314
	N1 ~ N3	245	7.890 ± 1.486	
M	M0	187	8.076 ± 1.488	0.153
	M1	1		
Stage	I–II	115	8.174 ± 1.267	0.300
	III–IV	390	8.010 ± 1.559	
Histologic grade	G1–G2	368	7.841 ± 1.410	0.001
	G3–G4	130	8.413 ± 1.622	
Lymphovascular invasion	Yes	122	7.988 ± 1.451	0.282
	No	229	7.814 ± 1.424	
Alcohol	Yes	346	8.065 ± 1.474	0.366
	No	164	7.936 ± 1.563	

### Comprehensive meta‐analysis based on microarrays

#### MiR‐99a‐5p expression level in HNSCC

**Figure 4:**
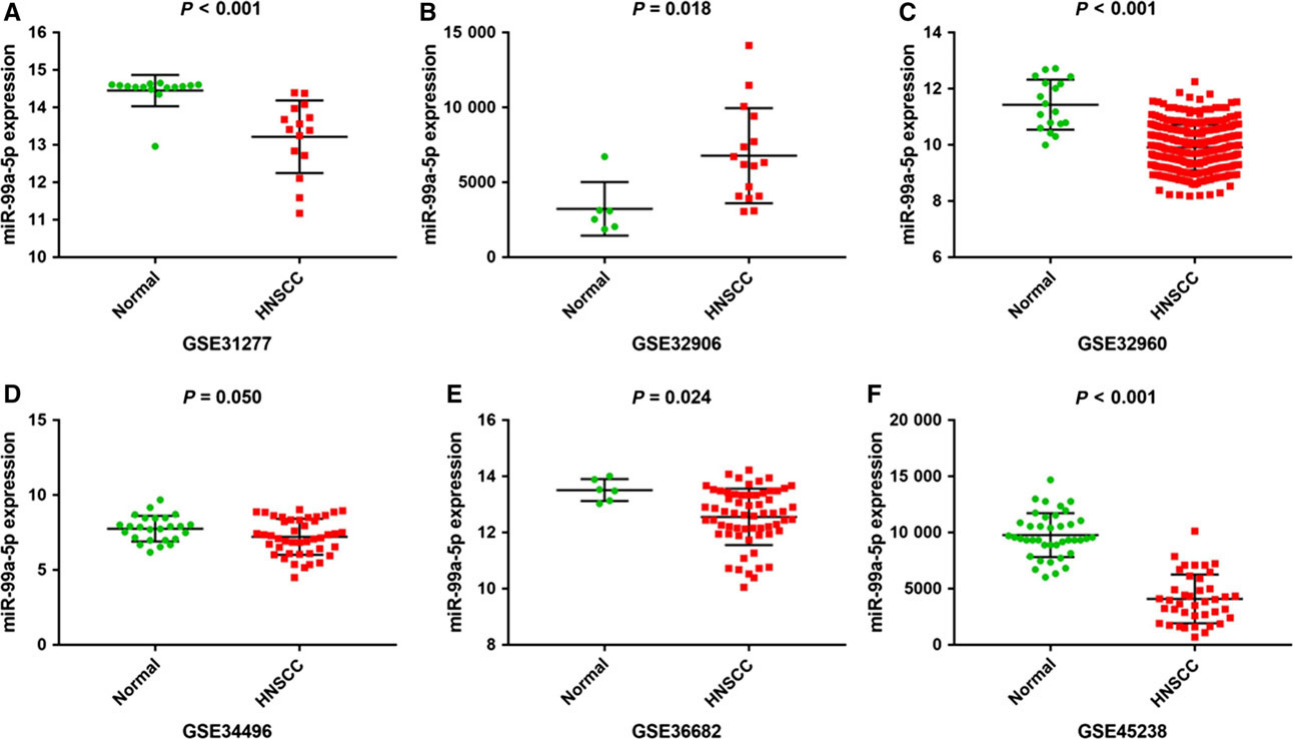
Representative scatter plots of miR‐99a‐5p expression data in normal and HNSCC tissues in microarrays. Expression data of miR‐99a‐5p in normal and HNSCC tissues from microarrays with *P* value ≤ 0.05 were plotted: (A) http://www.ncbi.nlm.nih.gov/geo/query/acc.cgi?acc=GSE31277 (*P* < 0.001). (B) http://www.ncbi.nlm.nih.gov/geo/query/acc.cgi?acc=GSE32906 (*P* = 0.018). (C) http://www.ncbi.nlm.nih.gov/geo/query/acc.cgi?acc=GSE32960 (*P* < 0.001). (D) http://www.ncbi.nlm.nih.gov/geo/query/acc.cgi?acc=GSE34496 (*P* = 0.050). (E) http://www.ncbi.nlm.nih.gov/geo/query/acc.cgi?acc=GSE36682 (*P* = 0.024). (F) http://www.ncbi.nlm.nih.gov/geo/query/acc.cgi?acc=GSE45238 (*P* < 0.001).

**Figure 5:**
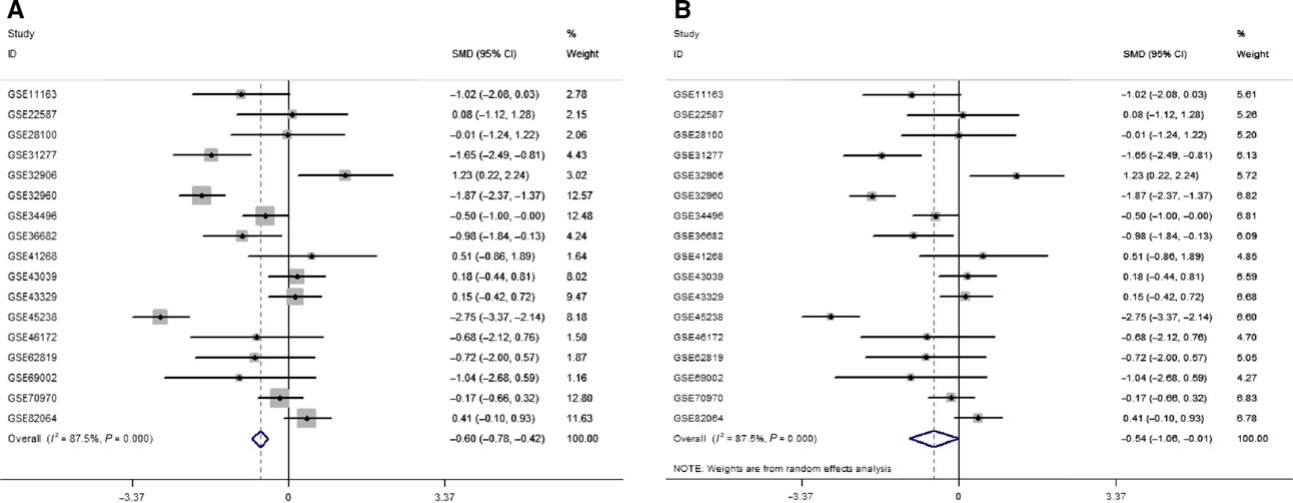
MiR‐99a‐5p expression levels in normal tissue and HNSCC by forest plot. (A) Forest plot constructed by the fixed‐effect model (SMD = −0.60, 95% CI= −0.78 to −0.42, *I* ^*2*^ = 87.5%). (B) Forest plot constructed by the random‐effect model (SMD = −0.54, 95% CI = −1.06 to −0.01, *I* ^*2*^ = 87.5%).

**Figure 6:**
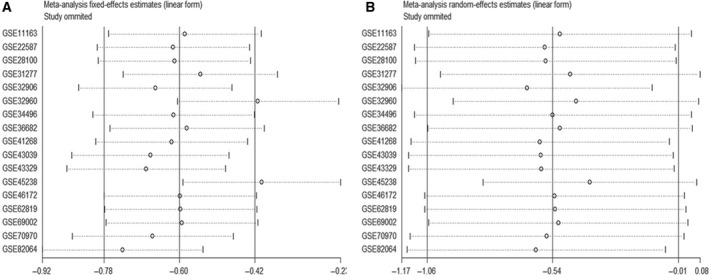
Sensitivity analysis. (A) Fixed‐effect model. (B) Random‐effect model. http://www.ncbi.nlm.nih.gov/geo/query/acc.cgi?acc=GSE45238 and http://www.ncbi.nlm.nih.gov/geo/query/acc.cgi?acc=GSE32960 might be sources of heterogeneity according to the fixed‐effect model.

**Figure 7:**
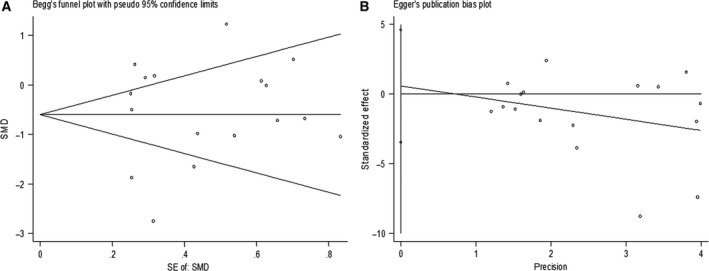
Publication bias detection. (A) Begg's funnel. (B) Egger's plot.

A total of 18 eligible microarrays were selected from GEO datasets. Finally, 924 HNSCC tissues and 212 noncancerous head and neck tissues were included as http://www.ncbi.nlm.nih.gov/geo/query/acc.cgi?acc=GSE34496 and http://www.ncbi.nlm.nih.gov/geo/query/acc.cgi?acc=GSE73460 acted equally. However, no publications met our criteria. Then, continuous variable meta‐analysis pooled the expression data from 17 microarrays (Table 3), among which there were 6 significant microarrays (*P* ≤ 0.05; Fig. 4). Other microarrays without statistical significance were displayed in Fig. S1 (A‐K). As the overall result revealed, miR‐99a‐5p expression was lower in HNSCC tissue than in the control group both for fixed‐effect (SMD = −0.60, 95% CI = −0.78 to −0.42, *I* ^*2*^ = 87.5%) and random‐effect (SMD = −0.54, 95% CI = −1.06 to −0.01, *I* ^*2*^ = 87.5%) models (Fig. 5). Sensitivity analysis was then carried out to evaluate the influence of each chip. http://www.ncbi.nlm.nih.gov/geo/query/acc.cgi?acc=GSE45238 and http://www.ncbi.nlm.nih.gov/geo/query/acc.cgi?acc=GSE32960 might be the sources of heterogeneity (Fig. 6). Additionally, Begg's funnel and Egger's plot indicated no obvious publication bias (Fig. 7).

**Table 3:** Basic characteristics and data of the included microarrays

Accession	Author	Year	Country	Platform	Sample	Exp mean ± Exp SD	Ctrl mean ± Ctrl SD	TP	FP	FN	TN
http://www.ncbi.nlm.nih.gov/geo/query/acc.cgi?acc=GSE11163	Avissar M *et al*.	2008	USA	GPL6680	21	8.952 ± 1.693	10.531 ± 0.792	12	1	4	4
http://www.ncbi.nlm.nih.gov/geo/query/acc.cgi?acc=GSE22587	Li T *et al*.	2013	China	GPL8933	12	560.969 ± 304.226	540.273 ± 73.556	3	0	5	4
http://www.ncbi.nlm.nih.gov/geo/query/acc.cgi?acc=GSE28100	Jung HM *et al*.	2012	USA	GPL10850	20	8.151 ± 1.449	8.167 ± 1.431	15	2	2	1
http://www.ncbi.nlm.nih.gov/geo/query/acc.cgi?acc=GSE31277	Severino P *et al*.	2014	Brazil	GPL4133	30	13.218 ± 0.97	14.45 ± 0.418	15	2	0	13
http://www.ncbi.nlm.nih.gov/geo/query/acc.cgi?acc=GSE32906	Luo Z *et al*.	2012	China	GPL11350	22	6780.977 ± 3169.49	3233.222 ± 1786.07	16	6	0	0
http://www.ncbi.nlm.nih.gov/geo/query/acc.cgi?acc=GSE32960	Ma J *et al*.	2012	China	GPL14722	330	9.913 ± 0.808	11.433 ± 0.888	242	3	70	15
http://www.ncbi.nlm.nih.gov/geo/query/acc.cgi?acc=GSE34496	Ochs MF *et al*.	2013	USA	GPL8786	69	7.215 ± 1.202	7.762 ± 0.858	27	8	17	17
http://www.ncbi.nlm.nih.gov/geo/query/acc.cgi?acc=GSE36682	Wei R *et al*.	2012	China	GPL15311	68	12.56 ± 1.001	13.513 ± 0.391	40	0	22	6
http://www.ncbi.nlm.nih.gov/geo/query/acc.cgi?acc=GSE41268	Xie Z *et al*.	2012	China	GPL10850	10	5.978 ± 0.995	5.499 ± 0.703	1	0	6	3
http://www.ncbi.nlm.nih.gov/geo/query/acc.cgi?acc=GSE43039	Li X *et al*.	2015	China	GPL16414	40	−0.053 ± 3.322	−0.515 ± 1.23	2	0	18	20
http://www.ncbi.nlm.nih.gov/geo/query/acc.cgi?acc=GSE43329	Zheng X *et al*.	2013	China	GPL16475	50	102.21 ± 30.963	98.606 ± 0.583	6	2	25	17
http://www.ncbi.nlm.nih.gov/geo/query/acc.cgi?acc=GSE45238	Shiah S *et al*.	2015	Taiwan	GPL8179	80	4094.715 ± 2167.8	9774.709 ± 1957.02	38	4	2	36
http://www.ncbi.nlm.nih.gov/geo/query/acc.cgi?acc=GSE46172	Plieskatt JL *et al*.	2014	USA	GPL16770	8	8.262 ± 2.384	9.443 ± 0.614	2	0	2	4
http://www.ncbi.nlm.nih.gov/geo/query/acc.cgi?acc=GSE62819	Lian M *et al*.	2014	China	GPL16384	10	10.288 ± 1.185	11.078 ± 1.012	4	1	1	4
http://www.ncbi.nlm.nih.gov/geo/query/acc.cgi?acc=GSE69002	Creighton C *et al*.	2016	USA	GPL18044	7	3.305 ± 0.058	3.405 ± 0.115	3	2	0	2
http://www.ncbi.nlm.nih.gov/geo/query/acc.cgi?acc=GSE70970	Bruce J *et al*.	2015	Canada	GPL20699	263	9.078 ± 2.033	9.421 ± 0.891	73	1	173	16
http://www.ncbi.nlm.nih.gov/geo/query/acc.cgi?acc=GSE82064	Valeri N *et al*.	2017	Switzerland	GPL21968	96	212.885 ± 194.605	139.667 ± 60.085	21	4	57	14

#### Diagnostic value of miR‐99a‐5p for HNSCC

**Figure 8:**
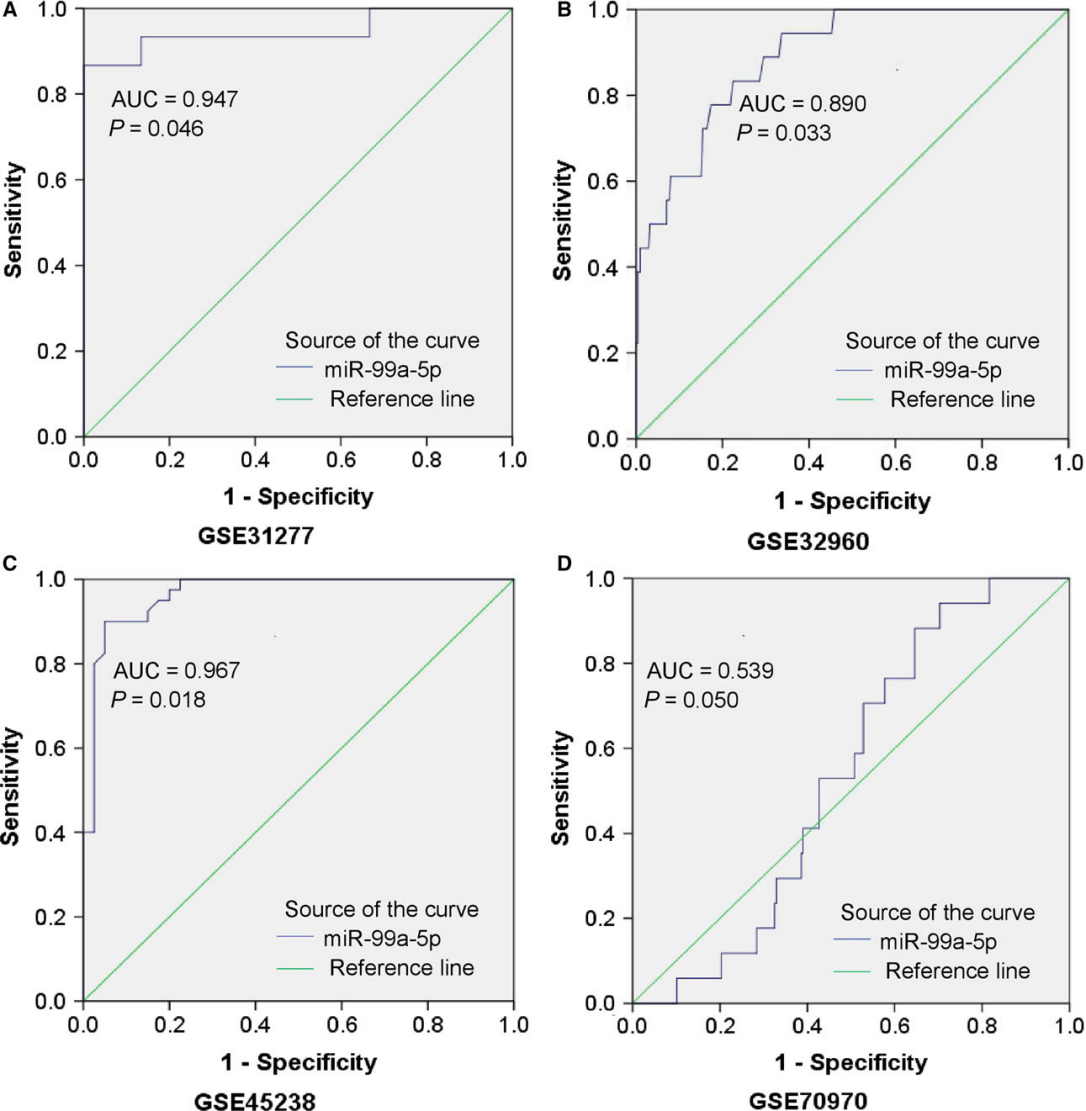
Representative ROC curves of the microarrays. ROC curve of miR‐99a‐5p expression in normal and HNSCC tissues from microarrays with *P* value ≤ 0.05 were plotted: (A) http://www.ncbi.nlm.nih.gov/geo/query/acc.cgi?acc=GSE31277 (AUC = 0.947, *P* = 0.046). (B) http://www.ncbi.nlm.nih.gov/geo/query/acc.cgi?acc=GSE32960 (AUC = 0.890, *P* = 0.033). (C) http://www.ncbi.nlm.nih.gov/geo/query/acc.cgi?acc=GSE45238 (AUC = 0.967, *P* = 0.018). (D) http://www.ncbi.nlm.nih.gov/geo/query/acc.cgi?acc=GSE70970 (AUC = 0.539, *P* = 0.050).

**Figure 9:**
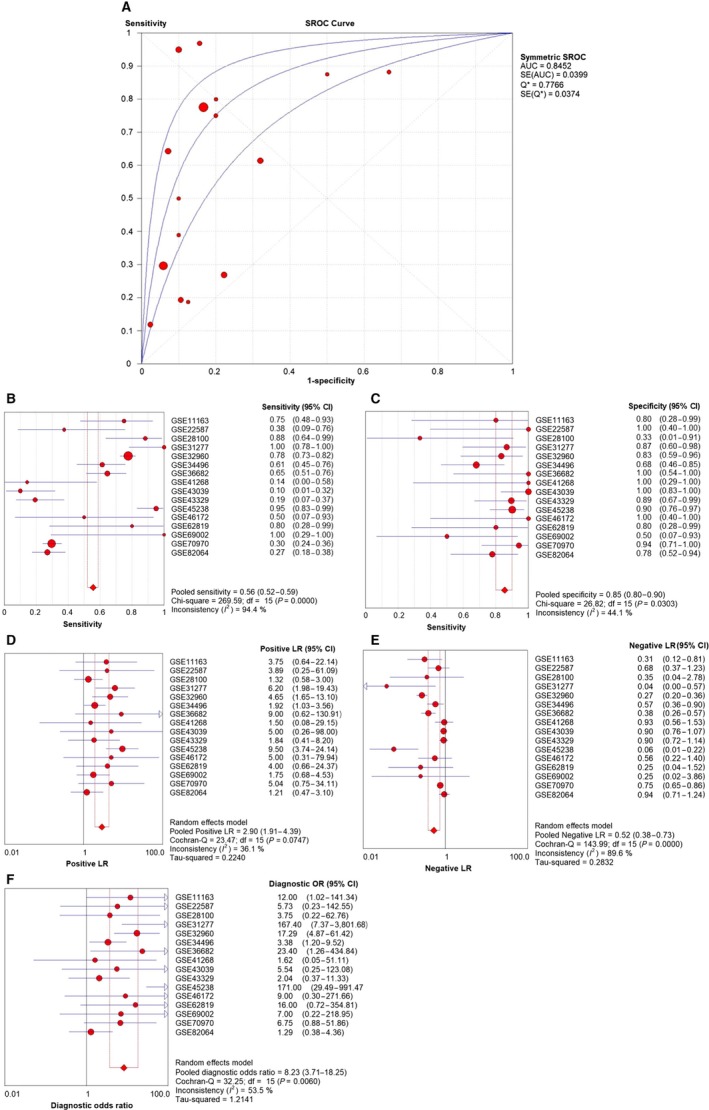
Diagnostic test based on the 17 included microarrays. (A) The summarized receiver operating characteristic (SROC) curve (AUC = 0.85, 95% CI = 0.77–0.92 calculated manually). (B) Sensitivity value of 0.56 (95%CI = 0.52–0.59, *P* < 0.001). (C) Specificity value of 0.85 (95% CI = 0.80–0.90, *P* = 0.030). (D) Pool positive likelihood ratio of 2.90 (95% CI = 1.91–4.39, *P* = 0.074). (E) Pool negative likelihood ratio of 0.52 (95% CI = 0.38–0.73, *P* < 0.001). (F) Diagnostic odds ratio of 8.23 (95% CI = 3.71–18.25, *P* = 0.006).

ROC curves for all eligible studies were plotted. Four representative ROC curves including http://www.ncbi.nlm.nih.gov/geo/query/acc.cgi?acc=GSE31277, http://www.ncbi.nlm.nih.gov/geo/query/acc.cgi?acc=GSE32960, http://www.ncbi.nlm.nih.gov/geo/query/acc.cgi?acc=GSE36682 and http://www.ncbi.nlm.nih.gov/geo/query/acc.cgi?acc=GSE45238 were displayed in Fig. 8, with *P* value less than 0.05, while the other studies showing no statistical significance were in Fig. S1 (L‐X). Further analysis was performed based on the TP, FP, FN, and TN results (Table 3). As shown in Fig. 9, the SROC curve verified the diagnostic value of miR‐99a‐5p in HNSCC as the AUC was 0.85 (95% CI = 0.77–0.92), with a sensitivity of 0.56 (95%CI=0.52–0.59, *P* < 0.001) and a specificity of 0.85 (95% CI = 0.80–0.90, *P* = 0.030). Furthermore, likelihood ratios were calculated (Pool +LR = 2.90, 95% CI = 1.91–4.39, *P* = 0.074; pool −LR = 0.52, 95% CI = 0.38–0.73, *P* < 0.001), respectively. The diagnostic OR of 8.23 (95% CI = 3.71–18.25, *P* = 0.006) also suggested a significant diagnostic value when based on all the included samples. However, only the http://www.ncbi.nlm.nih.gov/geo/query/acc.cgi?acc=GSE41268 and http://www.ncbi.nlm.nih.gov/geo/query/acc.cgi?acc=GSE69002 chips provided body fluid samples (AUC = 0.333, *P* = 0.201 and AUC = 0.750, *P* = 0.197, respectively). Thus, the clinical diagnostic value of miR‐99a‐5p for HNSCC could not be fully verified.

### Bioinformatics analyses of miR‐99a‐5p and HNSCC

#### Prediction of miR‐99a‐5p target genes

**Figure 10:**
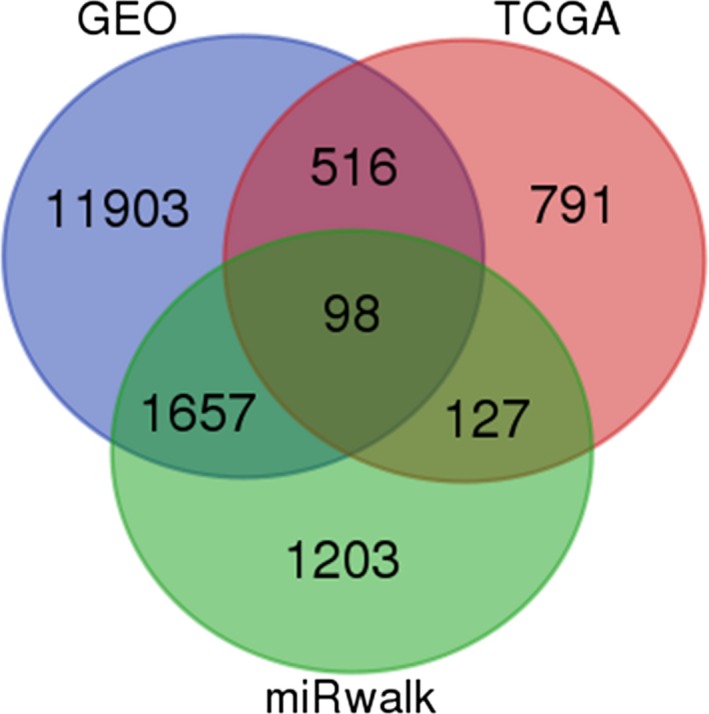
Venn diagram for identifying 98 promising target genes of miR‐99a‐5p in HNSCC.

We screened 14 174 genes from GSM2279805, 1532 genes from the TCGA dataset, and 3085 genes from miRwalk 2.0 after removing duplicates. As the analytical integration shown, a total of 98 genes overlapped in the GSM microarray and online software (Fig. 10). *MTMR3*,* IGF1R*,* MTOR*,* NOX4,* and *HOXA1*, which were searched from published literature, were also included 49, 50, 51, 52, 53. Finally, a total of 103 genes were identified as the promising target genes of miR‐99a‐5p in HNSCC (Table 4).

**Table 4:** Validated genes of miR‐99a‐5p from the GEO, TCGA, and miRwalk databases and literature

	Genes					
Validated in HNSCC	MTMR3	IGF1R	MTOR	NOX4	HOXA1	
Validated by analytical integration	LAMA5	AGO2	HIP1	SLC44A1	TNFAIP8L1	POLE
	RELB	U2SURP	RNF213	DNASE2	MPP3	TRAM2
	LHFPL2	NCS1	FAM64A	TTYH3	HSP90B1	GUCY1A3
	WNT7B	CELSR1	SLC39A14	MXRA8	NRIP3	PAPLN
	ASNS	PIK3CD	NAV1	FANCA	STK10	AFAP1L1
	B4GALNT1	TAPBP	WARS	TUSC3	CASK	PRSS23
	HENMT1	ACVR1	ANGPT2	BICD1	DLX5	RGS3
	ABCG1	ITGA3	SH2B3	COL5A1	CTLA4	ECE1
	ETS1	EXT2	FOXM1	BCAM	MN1	PDGFRB
	PYCR1	MAPK12	SLC1A4	GPR68	STC2	UBE2L6
	SCRN1	IGF2BP2	TSPAN9	SLC2A6	KIAA0930	FLRT2
	TMEM184B	DKK3	EPB41L4B	KIRREL	YEATS2	IPO9
	FAM111A	STRA6	ORAI2	LBH	APH1B	KRBA1
	RNASE7	MARVELD3	C1QTNF1	ANTXR2	GJB4	C10orf35
	NPNT	SH3PXD2B	ADAM8	COL6A2	HAS2	ODC1
	TGFB3	FADD	DDIT4	MISP	MFAP5	SYT7
	ARHGEF39	NRBP2				

#### GO enrichment and KEGG pathway analysis

**Figure 11:**
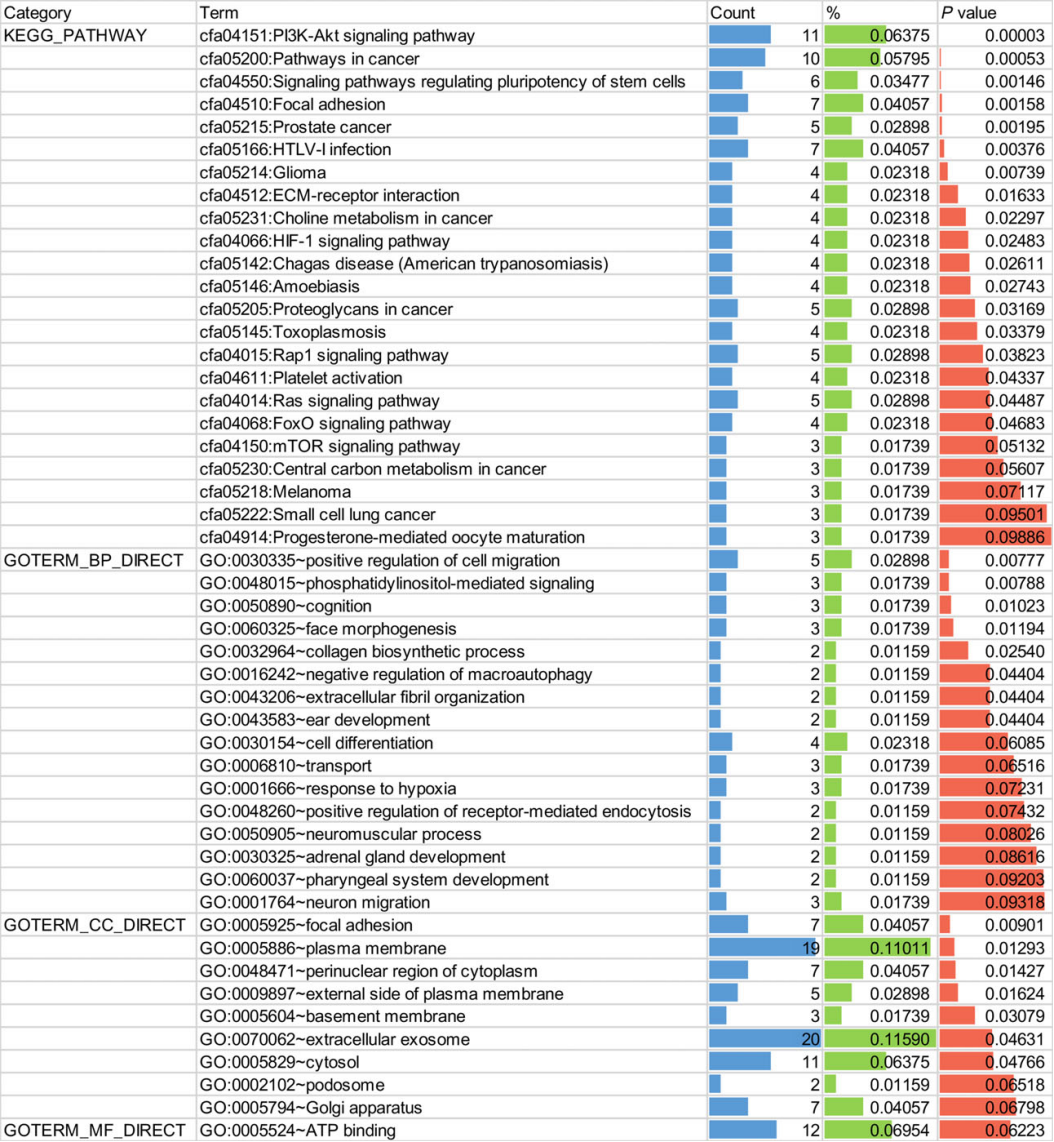
Functional annotation of target genes by GO enrichment and KEGG pathway analysis (*P* value < 0.05 for KEGG, BP, and CC).

**Figure 12:**
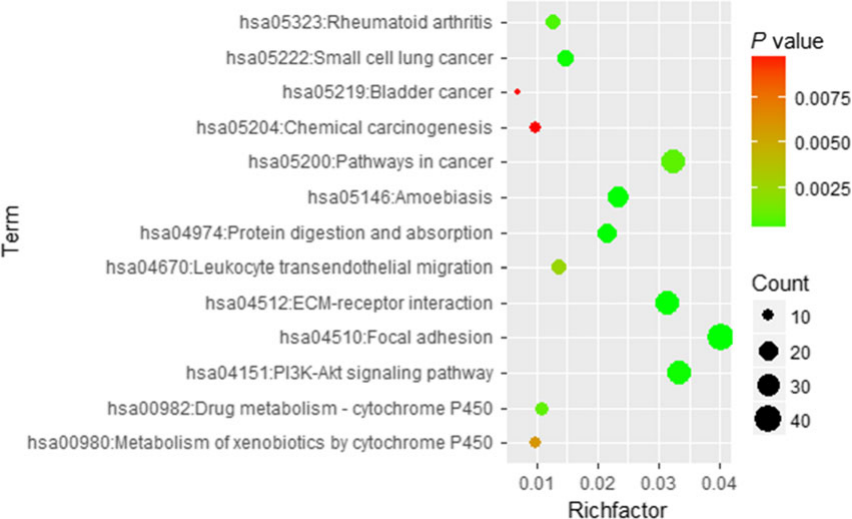
Bubble diagram of KEGG pathway for HNSCC. Significant pathways with *P* value < 0.01 were plotted by R language.

For GO enrichment analysis, the results comprised biological process (BP), cellular component (CC), and molecular function (MF). The potential target genes of miR‐99a‐5p significantly influence 15 GO terms (*P* < 0.05), including cell migration and phosphatidylinositol‐mediated signaling in BP, and focal adhesion in CC. Additionally, KEGG pathway analysis also indicated that the PI3K‐Akt signaling pathway and pathways in cancer were the most enriched for miR‐99a‐5p in HNSCC (*P* < 0.001, FDR < 0.05; Fig. 11). According to another KEGG pathway analysis, the PI3K‐Akt signaling pathway and pathways in cancer were also confirmed to be significant in the progression of HNSCC (*P* < 0.001; Fig. 12).

### PPI network construction

**Figure 13:**
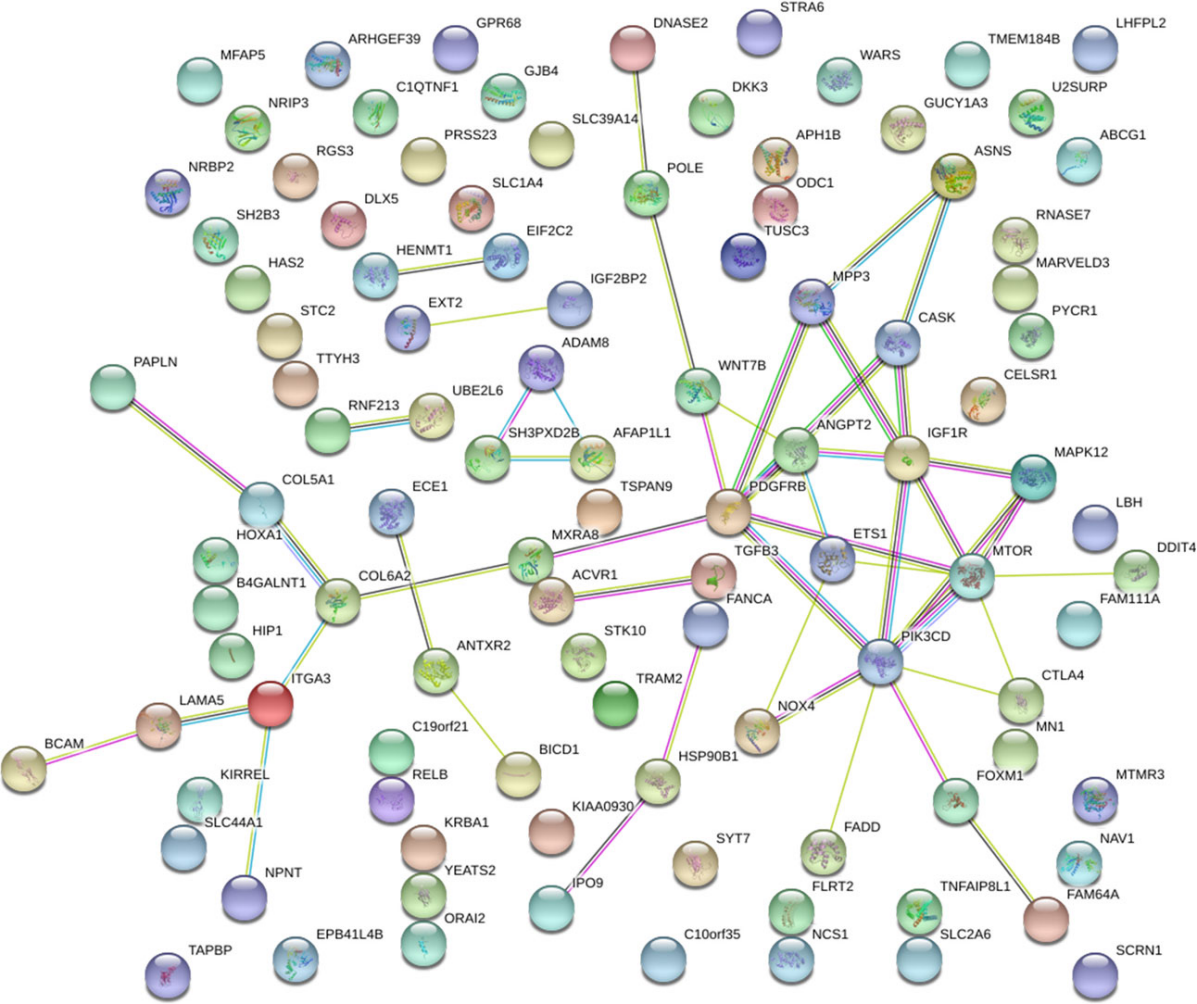
PPI network of 103 promising target genes of miR‐99a‐5p in HNSCC.

The 103 putative target genes were inputted into STRING for constructing a PPI network (Fig. 13). There were 103 nodes and 49 edges with an enrichment *P* value of 0.007. Thus, we further identified *PIK3CD*,* IGF1R*,* PDGFRB*, and *MTOR* as the hub genes of miR‐99a‐5p in HNSCC (all degrees > 5).

### Expression value of hub genes and their correlations with miR‐99a‐5p

**Figure 14:**
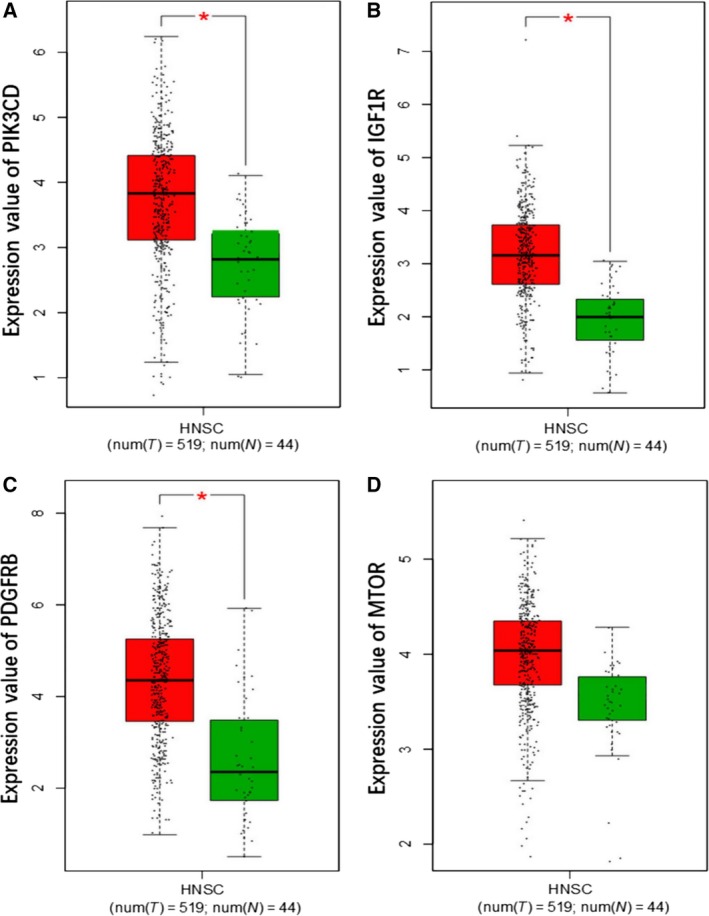
Expression analysis of four hub genes in 44 normal tissues and 519 HNSCC tissues based on GEPIA. (A) Expression value of *PIK3CD*. (B) Expression value of *IGF1R*. (C) Expression value of *PDGFRB*. (D) Expression value of *MTOR*.

**Figure 15:**
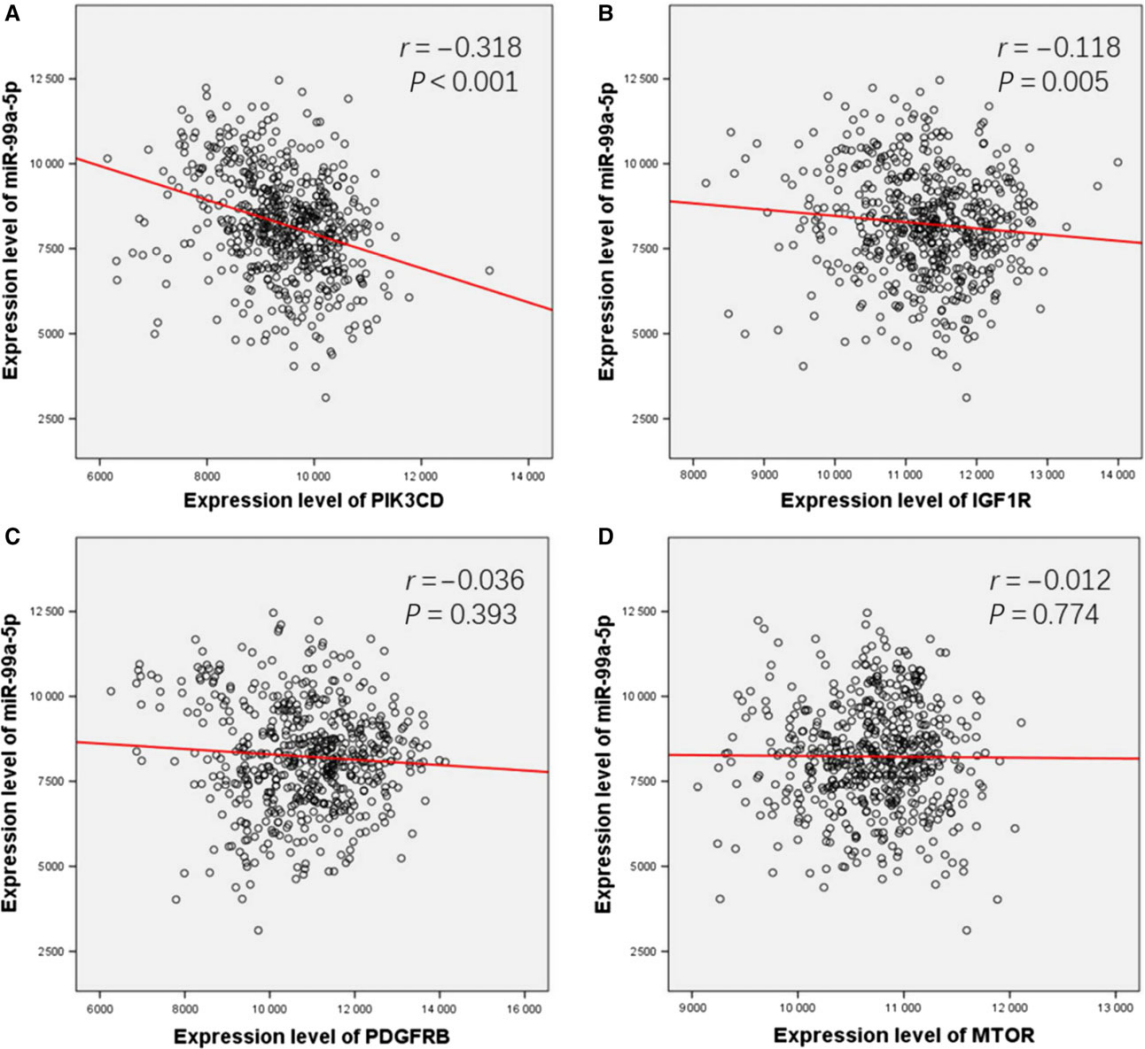
Spearman's correlation analysis. Expression value of *PIK3CD* (A), *IGF1R* (B), *PDGFRB* (C), and *MTOR* (D) and their correlations with miR‐99a‐5p.

As shown in the boxplot (Fig. 14), *PIK3CD*,* IGF1R*,* PDGFRB*, and *MTOR* all exhibited higher expression levels in 519 HNSCC tissues compared to the 44 normal tissues. *PIK3D* and *IGFR1* expression levels were significant negatively correlated to miR‐99a‐5p in HNSCC (PIK3D: *r* = −0.318, *P* < 0.001; IGFR1: *r* = −0.118, *P* = 0.005), while PDGFRB and MTOR were mildly negatively correlated with miR‐99a‐5p (PDGFRB: *r* = −0.036, *P* = 0.393; MTOR: *r* = −0.012, *P* = 0.774; Fig. 15).

## Discussion

There has been a trend of targeted treatment for human cancers in recent years. Illuminated by the abnormal biological signals in cancer cells, people commit to detecting novel biomarkers for exploring the potential mechanism of tumorigenesis and progression as well as further clinical applications such as cancer diagnosis, treatment, and prognosis. Therein, the miRNAs, which appear to be involved in gene regulation, were confirmed to be tumor suppressors or promoters 54, 55.

Several studies have demonstrated that miR‐99a‐5p could affect proliferation, migration, and invasion in various cancers, including HNSCCs, via modulating gene expression 36, 56, 57. The reduced miR‐99a‐5p expression was confirmed to suppress the insulin‐like growth factor mammalian of rapamycin signaling (IGF‐mTOR signaling) through binding sites in their 3ʹ‐untranslated regions (UTRs) in childhood adrenocortical tumors 58. According to Kuo *et al*., miR‐99a‐5p could inhibited myotubularin‐related protein 3 (MTMR3) expression then suppress the metastasis of oral cancer (OC) 49. As for HNSCC, Chen *et al*. also reported that IGFR1 and MTOR were repressed by ectopic transfection of miR‐99‐5p 51. In addition, Yan *et al*. suggested that downregulation of miR‐99a‐5p contributed to oral squamous cell carcinoma (OSCC) by targeting MTOR, further indicating the relation between miR‐99a‐5p and HNSCC development 59. Recently, miR‐99a‐5p was found to facilitate oral tumor cells by targeting NADPH oxidase 4 (NOX4) 52. To achieve a deeper understanding of the mechanism underlying miR‐99a‐5p, we identified its potential targets and performed the comprehensive biological pathway analysis. According to our KEGG pathway analysis, miR‐99a‐5p significantly affected the progression of HNSCC by regulating the PI3K‐Akt signaling pathway, of which the predicted target genes, phosphatidylinositol‐4,5‐bisphosphate 3‐kinase catalytic subunit data (*PIK3CD*), insulin‐like growth factor 1 receptor (*IGFR1*), platelet‐derived growth factor receptor, beta polypeptide (*PDGFRB*), and mechanistic target of rapamycin (*MTOR*) were involved.

The PI3K‐Akt signaling pathway, concretely explained as the phosphatidylinositol 3‐kinase (PI3K)/AKT/mammalian target of the rapamycin (mTOR) signaling pathway, is aberrant in many types of cancer 60, 61, 62, 63. The involved *PIK3CD*,* IGFR1*,* PDGFRB*, and *MTOR4*, also screened out by PPI construction, were further utilized to analyze the correlations with miR‐99a‐5p. We found that the four hub genes all exhibited higher expression levels in HNSCC tissues than in normal tissues, gaining more possibility to be the target genes of miR‐99a‐5p. Interestingly, a negative correlation was found between these four genes and miR‐99a‐5p, with *PIK3D* and *IGFR1* showing significant negative correlation with miR‐99a‐5p in HNSCC and *PDGFRB* and *MTOR* showing a mild negative correlation. Thus, together with the findings of previous researches and our results, we speculated that the dysfunctional PI3K‐Akt signaling pathway was implicated in the development of HNSCC. Moreover, it seems that PI3K‐Akt signaling pathway was regulated by miR‐99a‐5p according to the statistical correlation analysis, which further provided evidence for the potential clinical value of miR‐99a‐5p detection in HNSCC.

Statistical analysis of miR‐99a‐5p expression would confirm our speculation. Previous studies have suggested that repressed miR‐99a‐5p may contribute to tumorigenesis via being unable to control the target genes. Thus far, no study has specifically analyzed the miR‐99a‐5p expression level in HNSCC, but several studies have demonstrated the lower expression of miR‐99a‐5p in HNSCC 46, 51, 59. According to our GEO meta‐analysis and TCGA data mining results, the miR‐99a‐5p expression level was markedly lower in HNSCC than in normal tissues. In addition, miR‐99a‐5p expression was higher in low neoplasm histological grades than high histological grades, and the patient's age may also be a possible clinical parameter. Furthermore, we found that miR‐99a‐5p showed significance in diagnostic and prognostic tests; however, due to limited body fluid samples, the results could not be used as representative and its diagnostic applicability in the clinical setting could not be determined. Thus, additional studies are needed to demonstrate the clinical role of miR‐99a‐5p in the diagnosis and prognosis of HNSCC.

This study has some limitations. First, the different miRNA extraction methods may disturb the results of our meta‐analysis. Second, the HNSCC and corresponding samples were mostly derived from tissue sections, lead to an unverified diagnostic value. Third, although we utilized TCGA data to expand our data, the big gap between the quantity of cancerous and noncancerous tissues brought down the reliability. And fourth, the resource of online protein databases and relevant immunohistochemical staining samples were limited so that we could not further validate the function of miR‐99a‐5p via the protein level of hub genes. Despite these limitations, based on our meta‐analysis, the results suggest that miR‐99a‐5p expression was significantly lower in HNSCC than in normal tissue. Biological analysis also suggested that miR‐99a‐5p may participate in HNSCC by suppressing the hub genes, *PIK3CD*,* IGFR1*,* PNGFRB,* and *MTOR*.

In general, our study confirmed that miR‐99a‐5p might be a tumor suppressor in HNSCC with downregulated expression in HNSCC tissues, via the PI3K‐Akt signaling pathway. Further studies are required to elucidate the role of miR‐99a‐5p in diagnosis and prognosis for HNSCC and provide the basis for that miR‐99a‐5p execute its function via the protein level of the targets, especially those hub genes predicted by our research.

## Author contributions

YQ, KH, FW, and YF conceived and designed the study. YC and JY collected, extracted, and analyzed the data. YQ and KH guided the statistical process and ensured the necessary graphs. YC and JY wrote the manuscript. All authors read and approved the final manuscript.

## Supporting information


**Fig. S1.** (A‐K): Scatter plots of miR‐99a‐5p expression data in normal and HNSCC tissues in the other 11 microarrays without statistical significance. (L‐X): ROC curves of the other 13 microarrays without statistical significance.
