# Supplementary material for: Biological role and clinical value of miR‐99a‐5p in head and neck squamous cell carcinoma (HNSCC): A bioinformatics‐based study
Source: FEBS Open Bio. 2018 Jun 26;8(8):1280–98. doi: 10.1002/2211-5463.12478 (PMC6070648; doi:10.1002/2211-5463.12478)
Supplement: Supplementary file 1 — Fig. S1. (A‐K): Scatter plots of miR‐99a‐5p expression data in normal and HNSCC tissues in the other 11 microarrays without statistical significance. (L‐X): ROC curves of the other 13 microarrays without statistical significance. [file FEB4-8-1280-s001.pdf]

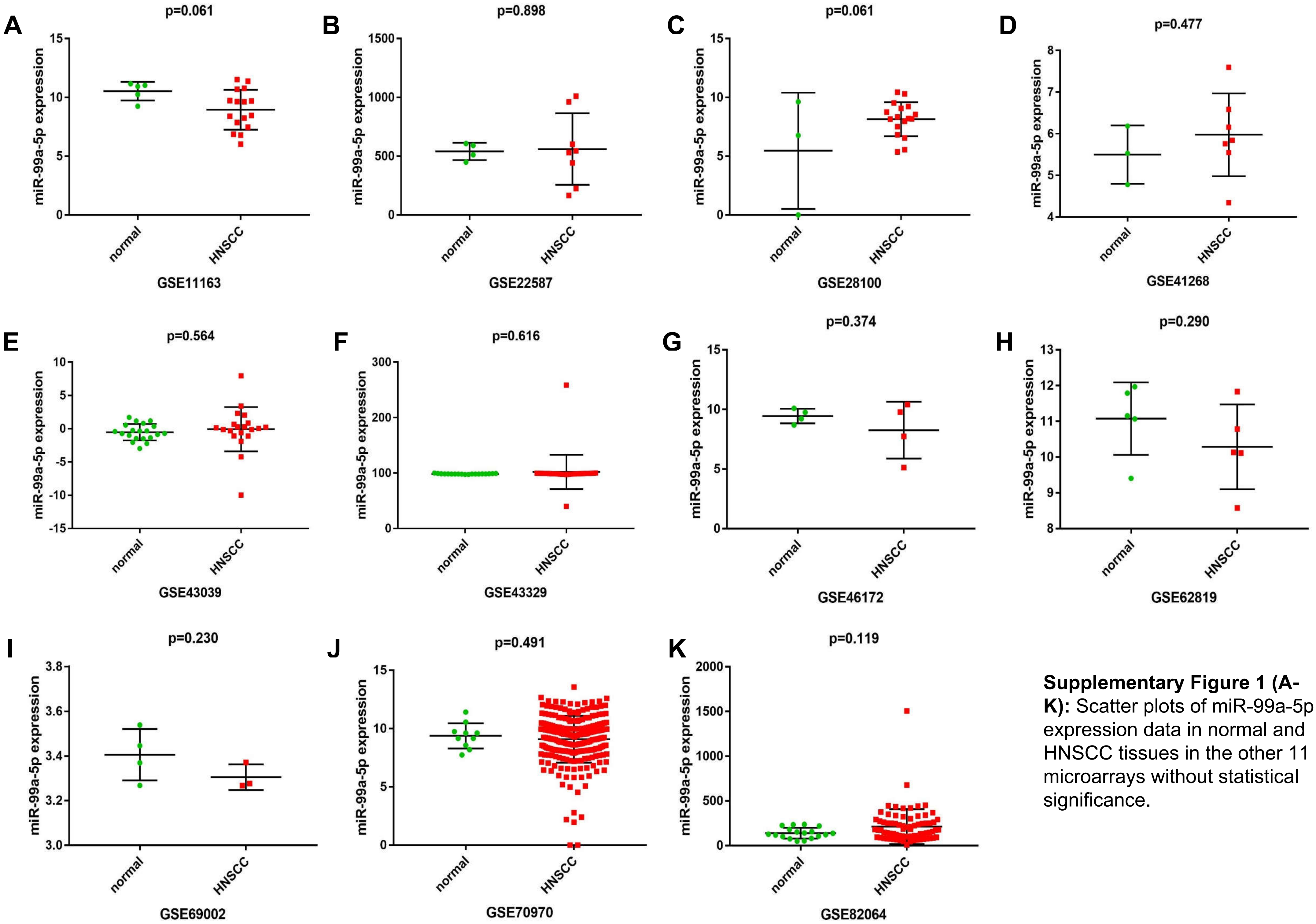

Supplementary Figure 1 (A-K): Scatter plots of miR-99a-5p expression data in normal and HNSCC tissues in the other 11 microarrays without statistical significance.

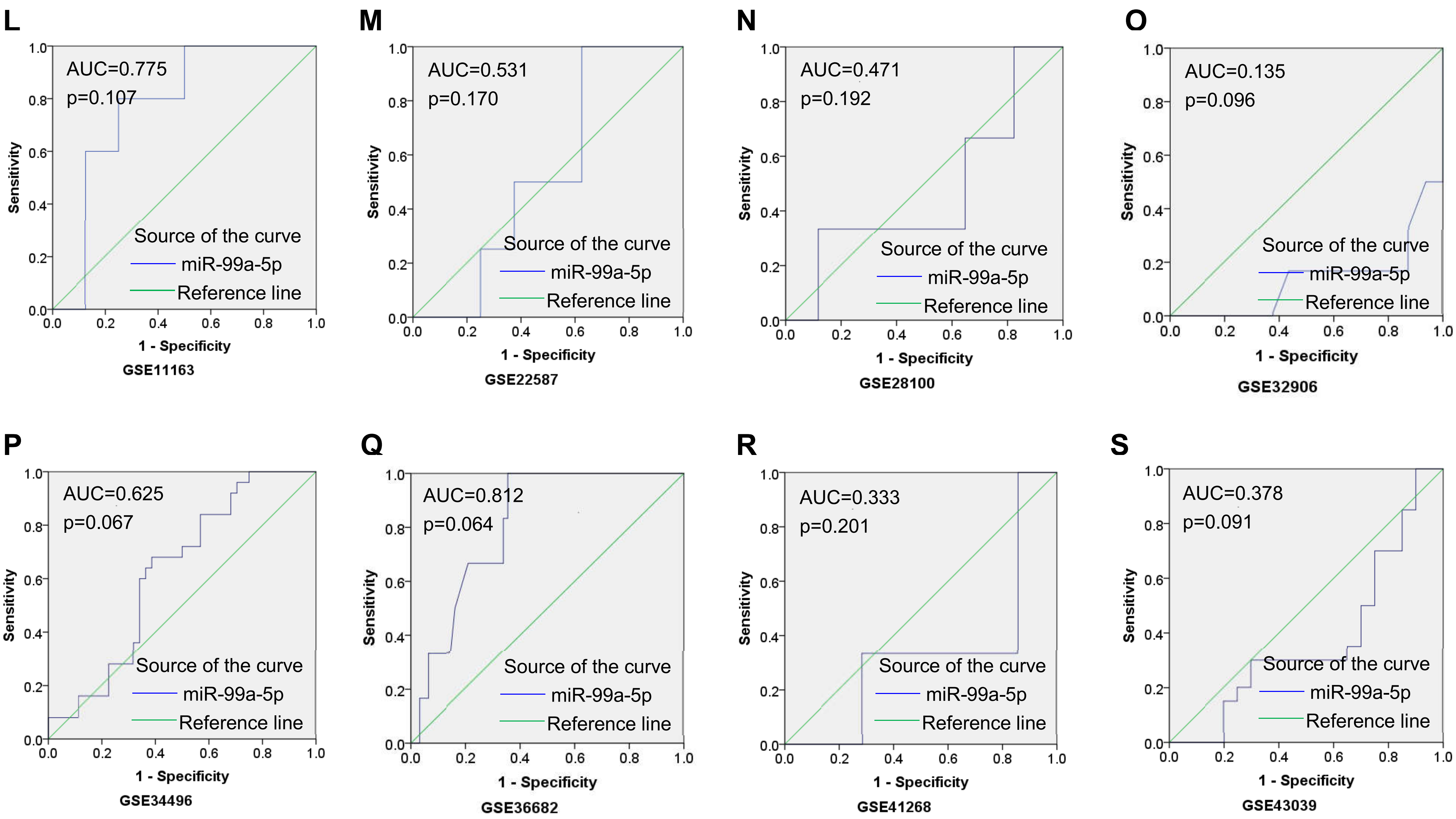

Supplementary Figure 1 (L-X): ROC curves of the other 13 microarrays without statistical significance**.**

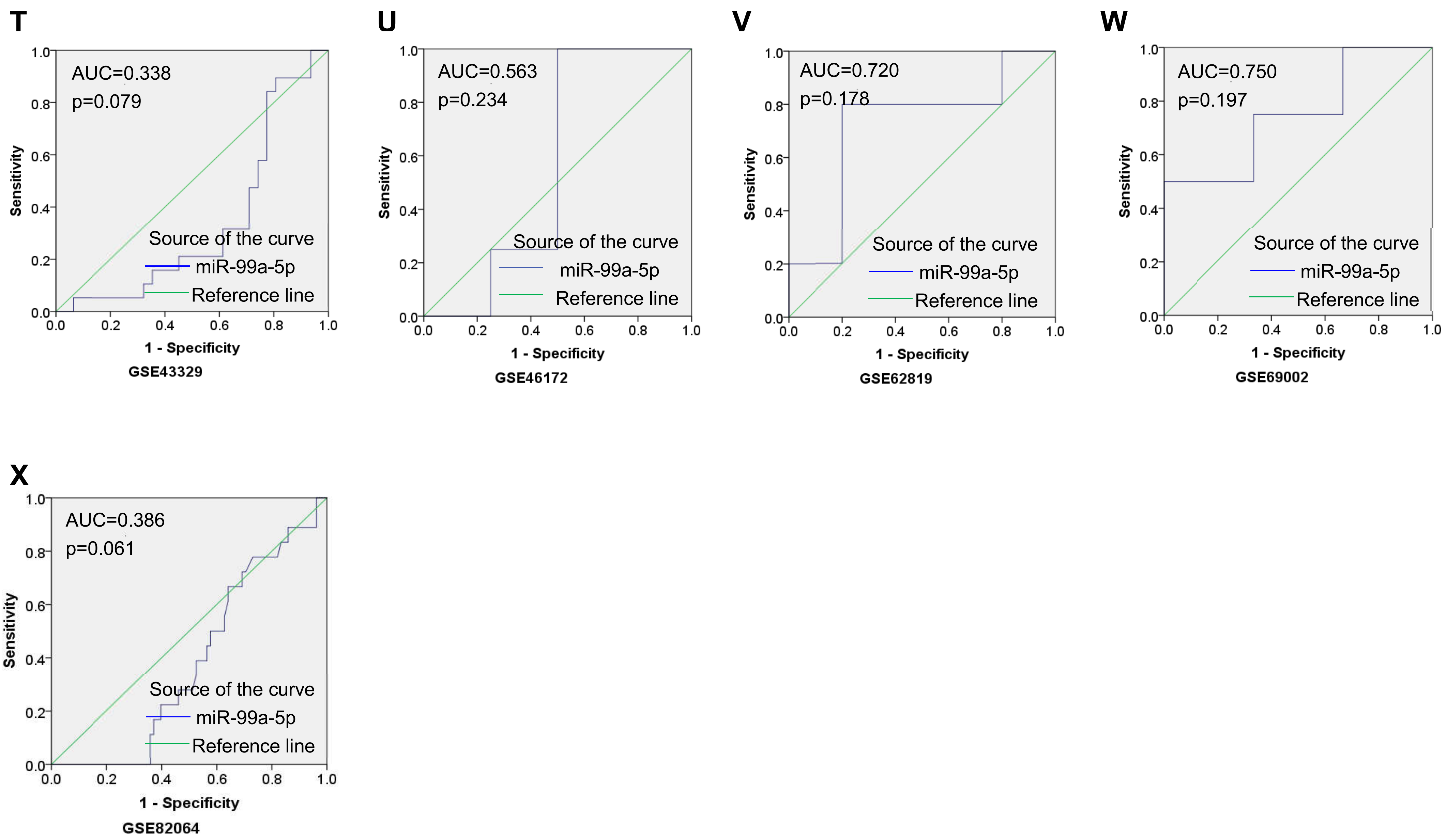

T
AUC=0.338
p=0.079
Source of the curve
miR-99a-5p
Reference line
Sensitivity
1 - Specificity
GSE43329
U
AUC=0.563
p=0.234
Source of the curve
miR-99a-5p
Reference line
Sensitivity
1 - Specificity
GSE46172
V
AUC=0.720
p=0.178
Source of the curve
miR-99a-5p
Reference line
Sensitivity
1 - Specificity
GSE62819
W
AUC=0.750
p=0.197
Source of the curve
miR-99a-5p
Reference line
Sensitivity
1 - Specificity
GSE69002
X
AUC=0.386
p=0.061
Source of the curve
miR-99a-5p
Reference line
Sensitivity
1 - Specificity
GSE82064
